# Physiological and Biochemical Response of Wild Olive (*Olea europaea* Subsp. *europaea* var. *sylvestris*) to Salinity

**DOI:** 10.3389/fpls.2021.712005

**Published:** 2021-08-30

**Authors:** Josip Tadić, Gvozden Dumičić, Maja Veršić Bratinčević, Sandra Vitko, Sandra Radić Brkanac

**Affiliations:** ^1^Department of Plant Sciences, Institute for Adriatic Crops and Karst Reclamation, Split, Croatia; ^2^Centre of Excellence for Biodiversity and Molecular Plant Breeding (CoE CroPBioDiv), Zagreb, Croatia; ^3^Department of Applied Sciences, Institute for Adriatic Crops and Karst Reclamation, Split, Croatia; ^4^Division of Botany, Department of Biology, Faculty of Science, University of Zagreb, Zagreb, Croatia

**Keywords:** wild olive, salinity, isotonic, osmotic stress, ion accumulation, lipid peroxidation, antioxidative enzyme

## Abstract

In the face of climate change, water deficit and increasing soil salinity pose an even greater challenge to olive cultivation in the Mediterranean basin. Due to its tolerance to abiotic stresses, wild olive (*Olea europaea* subsp. *europaea* var. *sylvestris*) presents a good candidate in breeding climate-resilient olive varieties. In this study, the early response of the native Croatian wild olive genotype (WOG) to salinity was evaluated and compared with that of well-known cultivars (cv.) Leccino and Koroneiki. Potted olive plants were exposed either to 150 mM NaCl or 300 mM mannitol for 3 weeks to distinguish between the osmotic and ionic components of salt stress. To determine the impact of the plant age on salinity, 1-, 2-, and 3-year-old WOG plants were used in the study. The growth parameters of both the cultivars and WOG of different ages decreased in response to the mannitol treatment. In contrast to cv. Leccino, the NaCl treatment did not significantly affect the growth of cv. Koroneiki or WOG of any age. The contents of Na^+^ and Cl^−^ were considerably higher in the salt-treated WOG, regardless of age, compared with the cultivars. However, while both treatments significantly reduced the K^+^ content of cv. Koroneiki, that nutrient was not significantly affected in either cv. Leccino or WOG. Unlike the cultivars and older WOG, the NaCl treatment caused a significant decline of photosynthetic pigments in the 1-year-old WOG. The cultivars and WOG of different ages experienced a similar drop in the chlorophyll *a* content under the isotonic mannitol treatment. The absence of lipid peroxidation, modulation of superoxide dismutase, and guaiacol peroxidase activity were noted in all WOG ages under both stressors. These data suggest that WOG resilience to salinity is associated with its large leaf capacity for Na^+^ and Cl^−^ accumulation, K^+^ retention, and its adaptable antioxidative mechanisms. The results are promising with regard to obtaining a new olive cultivar with better resilience to soil salinity.

## Introduction

The cultivation of agricultural crops is becoming increasingly challenged by the changing climate and a range of biotic and abiotic stresses (Brito et al., 2019). Among the latter, salinity is a major factor limiting the growth and production of plants (Urbanavičiute et al., 2021). Apart from primary salinization, extensive human activities such as irrigation can also increase soil salinity. Olive (*Olea europaea* subsp. *europaea* var. *europaea*) is one of the oldest domesticated trees in the Mediterranean basin (Lavee, 2013). Olive growing has changed from a traditional, extensive rain-fed crop to intensive or super-intensive orchards with high input demands (Lavee, 2013). Water deficit imposed by increased soil salinity as a consequence of irrigation with low-quality water (e.g., brackish and saline) remains the main restriction in olive cultivation in the Mediterranean basin (Vitagliano and Sebastiani, 2002; Chartzoulakis, 2005; Munns and Tester, 2008). The olive tree is moderately resistant to salinity and drought compared with other fruit trees, and this trait appears to be cultivar-dependent (Tattini et al., 1992; Loreto et al., 2003; Chartzoulakis, 2005; Perica et al., 2008; Masmoudi-Charfi et al., 2010). In response to the increased salinity, osmotic stress occurs instantaneously with a decrease in water potential (WP) and plant turgor, leading to the closure of the stomata and photosynthesis restriction (Munns, 2002). Later, salt-specific effects develop, although the intensity of stress depends on the concentration of Na^+^ and Cl^−^ ions and the duration of the stress (Chartzoulakis, 2005; Munns and Tester, 2008). Under typical conditions, the plant maintains a high K^+^/Na^+^ ratio in the cell cytosol, whereas Na^+^ maintains an osmotic balance in the growing tissue (Munns and Tester, 2008; Munns et al., 2019). In order to protect the shoots and young leaves, the olive tree preferentially stores salt ions in the roots; as the influx of salt ions continues, they are translocated to and accumulated in the stem and in old leaves (Chartzoulakis, 2005; Tattini and Traversi, 2009). Some degree of olive tolerance to elevated concentrations of Na^+^ and Cl^−^ in growing tissues is also determined by the ability to accumulate inorganic ions, primarily K^+^ and Ca^2+^ (Perica et al., 2008; Tattini et al., 2008). An increase of cytoplasmic Ca^2+^ could activate Ca^2+^-dependent protein kinases that can modify the affinity of the protein carrier from Na^+^ to K^+^ (Zhu, 2002) and participate in signaling pathways (Romeis et al., 2001). That pathway can include the biosynthesis of abscisic acid, which is partly responsible for the closure of the stomata (Wilkinson and Davies, 2002). High Ca^2+^ can also lead to a greater “osmotic imbalance,” but it can restrict the allocation of Na^+^ and Cl^−^ to sensitive shoots and shoot leaves. The advantage of olives over other fruit crops is their natural habitat in calcareous soils with available Ca^2+^ (Tattini and Traversi, 2009).

In addition to causing osmotic stress and ionic toxicity, salinity increases the generation of reactive oxygen species (ROS), which can lead to oxidative damage to macromolecules such as proteins, DNA, and membrane lipids (Das and Roychoudhury, 2014). To combat the oxidative stress, plants have developed a complex antioxidant defense system comprised of enzymatic components (e.g., superoxide dismutase [SOD], catalase, peroxidases, and glutathione reductase) and non-enzymatic components (e.g., ascorbic acid, tocopherol, and glutathione) (Hasegawa et al., 2000). Goreta et al. (2007) found that the assumed resistance of olives is determined by the period required for the plant to increase SOD concentrations in the tissue and observed that more sensitive olive genotypes accumulate higher concentrations of SOD over a shorter time, indicating earlier entry into oxidative stress.

Due to their high germplasm diversity (Belaj et al., 2011), shorter juvenile period of seedlings, and abundant flowering, wild (*O. europaea* subsp. *europaea* var. *sylvestris*) and feral olives are valuable reservoirs of genetic material that can be used in breeding programs for selection of new cultivars that can adapt to a changing climate (Leon et al., 2018). The aim of this study was to determine if the Croatian wild olive genotype (WOG) “Perišićeva mastrinka” is a potential candidate for a new olive cultivar with a higher resilience to increased soil salinity. We compared the WOG with the well-studied cultivars Koroneiki and Leccino, which are considered salt-sensitive and salt-resistant, respectively (Tattini, 1994; Chartzoulakis et al., 2002). Since salinity involves both osmotic and ionic stresses, we also aimed to discern between the effects of these two components to clarify the salt stress mechanism in the WOG. Thus, the objective was to investigate the early responses of Croatian WOG to salt stress by focusing on the discrimination between ionic and osmotic components of salinity. To evaluate whether the plant age has an influence on the adaptive response of the WOG to salinity, the WOG of different ages was included. The parameters such as the plant morphology, ionic relations, proline content, photosynthetic pigments, malondialdehyde (MDA), and enzyme activity [i.e., SOD and guaiacol peroxidase (GPOX)] were also examined in this context. Based on the results of this study performed under controlled conditions, the experiments will continue in the field, as the response of the WOG to salinity could be different.

## Materials and Methods

### Plant Material Collection and Rooting Process

The experiment was conducted with two well-known olive cultivars, namely, Leccino and Koroneiki, and WOG plants known as “Perišićeva mastrinka” of different ages (i.e., 1–3 years) in greenhouse conditions at the Institute for Adriatic Crops (IAC), Split (43°30′17.17″N, 16°29′49.71″E) in spring–summer 2019. Cuttings for the cultivars and WOG were obtained from the olive field collection at IAC (43°30′20.4″N, 16°29′54.0″E) and from a single 1,500-year-old olive tree from the town of Kaštela (43°33′02.0″N, 16°20′08.6″E). The WOG cuttings were collected over three consecutive years (October 2016, 2017, and 2018) to obtain specimens of different ages, including 1-year-old (WOG 1y), 2-year-old (WOG 2y), and 3-year-old (WOG 3y) plants. Leccino and Koroneiki cuttings were collected in October 2017.

Cuttings of 10 to 15 cm in length with one to two leaves were completely immersed in systemic fungicide Zino (Ningbo Synagrochem Co., Ltd., China) and left to dry until the fungicide gets absorbed. During the drying time, a solution (2,500 ppm) containing redistilled water, 96% ethyl alcohol, and indole-3 butyric acid (Sigma-Aldrich, St. Louis, United States) was prepared to stimulate the rooting process. The basal part of the cuttings was immersed in this solution for 10 s and allowed to dry completely. After drying, the cuttings were placed in a mist propagation system on a heated rooting table filled with a 20-cm layer of Agrilit 3 perlite (Perlite Italiana S.r.l., Corsico, Italy). The cuttings were inserted into the perlite to a depth of 3–5 cm. The rooting process lasted up to 3 months, and successfully, the rooted cuttings were then transplanted into small containers (9 cm) in a mixture of Agrilit 3 perlite and Brill TYPical 4 substrate (Brill Substrate GmbH & Co. KG, Georgsdorf, Germany) in a ratio of 1:1 (v:v). After 3 months of acclimatization, the young plants were again transplanted into 5-L pots in a mixture of Brill TYPical 4 substrate, Agrilit 3 perlite, and eutric brown soil (pH 5.5–6.8, humus 2–6%) in a ratio of 2:1:2 (v:v) and placed in an unshaded greenhouse.

### Growth and Experimental Conditions

Following a 1-year development period in the greenhouse, plants were removed from the pots, washed, and then transplanted to 3.6-L plastic pots filled with Agrilite 3 perlite (Perlite Italiana SRL., Milano, Italy) and vermiculite (RHP, Gravenzande, the Netherlands) substrate at a 1:1 (v:v) ratio. To prevent inorganic substrate leakage, 350 ml expanded clay (Laterlite S.P.A, Milano, Italy) was placed in the bottom of the pots. The acclimatization period was conducted in the greenhouse under a natural photoperiod from March to June 2019. During that period, minimal and maximal daily temperatures inside the greenhouse were 16.1°C and 32.2°C, respectively.

The plants were trimmed to one shoot and irrigated daily with half-strength Hoagland nutrient solution ($\frac{1}{2}$ HNS) (Hoagland and Arnon, 1950). The solution was prepared in a tank manually by diluting 100 × concentrated solution in tap water (1-L full-strength HNS was added to 100 L tap water). Furthermore, 0.1 M sulfuric acid was used to achieve the target pH of 5.5–6.5. The leaching fracture of 20–30% was analyzed daily to control pH (Mettler Toledo MP 230, Columbus, OH, United States) and electrical conductivity (EC) (Mettler Toledo MC 226, Columbus, OH, United States). A self-compensating emitter (Toro, Bloomington, MN, United States) was used for uniform irrigation of each plant with uniform amounts of HNS. Three polyethylene tanks Elbi CP 2000 [Elbi (Suisse) Sagl, Biasca, Switzerland] were used to store the irrigation solutions. The automatic control system (Schneider Electric, Rueil-Malmaison, France) enabled each treatment to have a separate irrigation regime and controlled indoor temperature with side and roof ventilation. The number of irrigations per day was adjusted by analyzing and sustaining the leaching fracture at 20–30% from the previous day. The experimental design was a randomized block experimental design with three replicates. The spatial arrangement of plants inside the greenhouse was east–west (Supplementary Figure 1).

After the 3-month acclimatization period, the experiment was started by exposing olive plants to 150 mM NaCl (non-iodized salt; Solana Pag d.d., Pag, Croatia) and 300 mM mannitol (powder; Roquette, Lestrem, France), which were added to $\frac{1}{2}$ HNS. To avoid osmotic shock, isosmotic concentrations of NaCl (−0.83 MPa) and mannitol (−0.82 MPa) were achieved gradually over a 3-day period with the daily increase of NaCl and mannitol by 50 and 100 mM, respectively. Control plants were irrigated with $\frac{1}{2}$ HNS only. After 3 weeks, the experiment was terminated. The greenhouse was then slightly shaded to avoid high temperatures during the day. During the experiment, the temperature in the greenhouse ranged from 25.7°C to 40.8°C, with an average of 24.08°C at night and 34.39°C during the day.

Physiological and biochemical parameters were measured in the young leaves, while the analyses with old leaves will be included in the next work.

### Morphometric and Physiological Measurements

Morphometric measurements were performed three times during the experiment, namely, on the first day, 12th day, and last day of the experiment. The last morphometric measurements were performed in the laboratory where the plants were cleaned of inorganic substrates and split into root, stem, and leaf. The following analyses included the measurement of (a) leaf and root dry weight (DW), (b) shoot length, (c) shoot area, and (d) shoot diameter. For the analysis of DW, the plant material was dried (Kambic Laboratory Equipment d.o.o., Semic, Slovenia) at 75°C for 48 h. The shoot length and shoot diameter were measured using a vernier caliper (Insize Co., Ltd, Suzhou New District, China), while the shoot area was determined using the WinFolia software package (Regent Instruments Inc., Québec, Canada).

Water potential (WP), electrolyte leakage (EL), and potassium leakage (KL) were determined in fresh samples 2 days before the end of the experiment. The WP was determined by taking a fully developed leaf from the top shoot of each sample using the PMS 1000 WP measuring device (Model 1000 Pressure Chamber, PMS Instrument Company, Oregon, SAD). By gradually increasing the pressure, the appearance of water droplets on the stem surface was monitored. When a droplet of water appeared, the current applied pressure (in bar) of nitrogen in the chamber was recorded, which determines the WP of the sample. The EL and KL were measured by taking a fully developed leaf from the shoot tip of each sample and cutting a 5-mm-diameter disk from the leaf and placing it in a glass vial. Vials were then filled with 30-ml redistilled water and left in a dark room for 24 h. EC1 and KL1 in all samples were measured using an EC meter MC 226 (Mettler-Toledo international Inc., OH, United States) or Sherwood 410 flame spectrometer (Sherwood Scientific Ltd, Cambridge, United Kingdom), respectively. Samples were then left in the same vials and autoclaved in the Presoclave II 80 (J.P. Selecta, Abrera, Spain) instrument at 120°C, 103.1 MPa for 20 min, and left for 24 h in a dark room to cool. EC2 and KL2 were measured for the second time.

Electrolyte leakage and PL were calculated according to the following expressions:

$$\begin{array}{lll} EL & = & EC1/\left(EC1+EC2\right)\times \;100\\ KL & = & KL1/\left(KL1+KL2\right)\times \;100 \end{array}$$

### Ion Content in Olive Leaves and Roots

The contents of calcium (Ca^2+^) and magnesium (Mg^2+^) were determined by atomic absorption spectrophotometry (AAS). The leaf and root samples were dried at 75°C for 48 h and then milled into a fine powder. For each sample, 1 g dry organic matter (± 0.001 g) was transferred in pots to the laboratory furnace at a temperature of 550–600°C for 60 min. After cooling, the samples were transferred to flasks and digested in HCl (30% v/v) solution. The solutions were then mixed and heated over open flame to the boiling point. The cooled solutions were filtrated using a 0.22-μm filter paper into 50-mL volumetric flasks, and deionized water was added to the mark of 50 ml. The contents of Ca^2+^ and Mg^+^ were then determined by using SpectraAA 220 AAS (Varian Inc., Palo Alto, CA, United States).

The contents of sodium (Na^+^), potassium (K^+^), and chloride (Cl^−^) in leaves were determined by using the Dionex DX500 ion chromatograph (Dionex Corporation, Sunnyvale, CA, United States). The samples (0.1 g) were added in Erlenmeyer flasks and filled with ultrapure water to a final volume of 25 mL. The samples were then placed in an ultrasonic bath for 30 min at 50°C and centrifuged for 5 min at 5,000 rpm. Prior to the measurement, solutions were filtered through 0.22- and 0.45-μm Polyethersulfone filters (PES). The analyses were performed using the PeakNet software package (Dionex Corporation).

### Biochemical Parameters

The first four fully developed leaves were sampled from the top of each shoot, which were sealed in a polyvinyl chloride bag and immersed in liquid nitrogen for several seconds. The samples were lyophilized (Labconco FreeZone 2.5 lyophilizer, Labconco Corporation, Kansas City, MO, United States) for 5 days after which the samples were stored at −65°C until the analysis. The leaves were homogenized using the IST400 mixer mill (InSolido Technologies, Zagreb, Croatia) for 1 min. For the determination of photosynthetic pigments, powdered samples (15 mg) were additionally homogenized in 80% (v/v) acetone for 1 min, and the contents of chlorophyll *a* (Chl *a*) and chlorophyll *b* (Chl *b*), and carotenoids (Car) were estimated according to Wellburn (1994). The contents of photosynthetic pigments were expressed as mg/g DW. For the analysis of antioxidative enzymes, the powdered material (50 mg) was additionally homogenized in potassium phosphate buffer (pH 7.0) for 1 min. The homogenates were centrifuged (Sigma 3K18 centrifuge; Osterode am Harz, Germany) at 25,000 g for 30 min at 4°C, and the supernatants were examined for enzyme activity and soluble proteins (Bradford, 1976). The protein contents of the enzyme extracts were determined using bovine serum albumin (Sigma-Aldrich) as a standard and expressed as mg/g DW. The activity of SOD was determined by measuring the ability of the enzyme to inhibit the reduction of nitroblue tetrazolium (Sigma-Aldrich) by superoxide (Beauchamp and Fridovich, 1971). For the calibration curve, bovine SOD was used as a standard. The activity of GPOX was measured using guaiacol as a substrate according to Chance and Maehly (1955). The formation of tetraguaiacol was monitored at 470 nm and quantified by taking its extinction coefficient (26.6 per mM/cm) into account. Specific enzyme activities were expressed as units/mg protein. The contents of MDA (an indicator of lipid peroxidation) and proline were determined according to Radić et al. (2009). The MDA content was measured using the thiobarbituric acid method at 532 nm and was calculated based on an extinction coefficient of 155 per mM/cm and expressed as nmol/mg DW. The proline content was estimated using the ninhydrin reagent from a calibration curve using l-proline (Sigma-Aldrich) as a standard, and the absorbance was read at 520 nm. The proline content was expressed as mg/g DW.

### Statistical Analysis

The statistical analysis was performed using the STATISTICA 13.3 package (TIBCO Software Inc., Palo Alto, CA, United States). The normality of data was tested by using the Shapiro–Wilk's test. The homogeneity of variance for each dependent variable was tested by using the Levene's test. For adequate comparison of olive cultivars and WOG, all data were normalized to set all controls to value 1. Differences between samples were assessed by using the one-way analysis of variance followed by the Duncan *post-hoc* comparison test. In all the statistical tests, the significance level was set to (*p* < 0.05). The original data are presented in Supplementary Tables 1–3.

## Results

### Morphometric and Physiological Parameters—Olives Are More Sensitive to Osmotic Stress Induced by Mannitol Than NaCl

The values of Water potential (WP) (ψ) and electrical leakage (EL) are displayed in Table 1. In comparison with the control values, WP of all olive genotypes dropped under the NaCl treatment, though the greatest decrease was observed under the mannitol treatment.

**Table 1:** Water potential (Ψ) and electrical leakage (EL).

**Genotype**	**Treatment**	**Ψ (bar)**	**EL (%)**
Koroneiki	Control	−14.7 (2.78)^d^	16.0 (0.87)^ab^
	NaCl	−22.2 (2.75)^b^	15.7 (3.62)^ab^
	Mannitol	−35.7 (3.79)^a^	16.3 (0.88)^ab^
Leccino	Control	−21.7 (3.74)^bc^	16.2 (2.79)^ab^
	NaCl	−31.3 (5.13)^a^	18.4 (2.62)^ab^
	Mannitol	−37.7 (4.95)^a^	17.5 (2.63)^ab^
WOG 1y	Control	−15.0 (2.46)^d^	14.3 (0.87)^b^
	NaCl	−23.3 (3.04)^b^	19.1 (1.63)^ab^
	Mannitol	−34.0 (1.02)^a^	17.6 (0.52)^ab^
WOG 2y	Control	−15.5 (2.77)^cd^	16.4 (3.17)^ab^
	NaCl	−24.7 (3.51)^b^	18.6 (4.31)^ab^
	Mannitol	−35.7 (3.21)^a^	20.19 (1.95)^a^
WOG 3y	Control	−13.7 (2.75)^d^	16.7 (4.19)^ab^
	NaCl	−23.8 (1.61)^b^	16.7 (3.62)^ab^
	Mannitol	−32.0 (1.73)^a^	19.1 (1.05)^ab^

*Data are averages of three replicates (SD in parenthesis). Different letters within the column present significant values at p < 0.05*.

An increase in electrical leakage accompanied plant response to both abiotic stressors (Table 1). The exception was the 3-year-old WOG, where a greater electrical leakage was recorded in the control than in the NaCl treatment.

Growth inhibition is the most common indicator of salinity stress in olive plants (Table 2; Supplementary Table 1). The mannitol treatment had a greater impact on the parameters of shoot growth than the NaCl treatment. The latter stressor did not significantly change the growth of cv. Koroneiki (except for shoot DW) or WOG of different ages, while most growth parameters were significantly reduced in the NaCl-treated cv. Leccino. On the other hand, the mannitol treatment caused a marked reduction of the parameters of shoot growth in all genotypes. Concerning the effect of age on the growth of the WOG, it is evident that both stressors reduced growth to a lesser degree with increasing age. Irrespective of the stressor, shoot diameter changed the least. Compared with shoot DW, root DW was generally less affected by mannitol. This parameter was markedly decreased in cv. Koroneiki and Leccino at similar levels under both stressors. On the other hand, the root DW of treated WOG did not significantly change compared with the control.

**Table 2:** The relative growth parameters measured in olives.

**Genotype**	**Treatment**	**Shoot increase (cm)**	**Shoot DW (g)**	**Shoot area (cm^**2**^)**	**Shoot diameter (mm)**	**Root DW (g)**
Koroneiki	Control	1.00 (0.365)^a^	1.00 (0.273)^a^	1.00 (0.311)^a^	1.00 (0.049)^a^	1.00 (0.178)^ab^
	NaCl	0.91 (0.225)^a^	0.66 (0.225)^bcd^	0.72 (0.194)^ab^	0.81 (0.044)^bc^	0.71 (0.076)^de^
	Mannitol	0.53 (0.112)^cd^	0.31 (0.123)^e^	0.29 (0.093)^d^	0.65 (0.056)^d^	0.75 (0.191)^cde^
Leccino	Control	1.00 (0.155)^a^	1.00 (0.211)^a^	1.00 (0.229)^a^	1.00 (0.183)^a^	1.00 (0.197)^ab^
	NaCl	0.69 (0.068)^bc^	0.48 (0.055)^cde^	0.65 (0.187)^bc^	0.94 (0.032)^a^	0.62 (0.019)^e^
	Mannitol	0.56 (0.080)^cd^	0.46 (0.021)^cde^	0.37 (0.070)^cd^	0.85 (0.056)^abc^	0.72 (0.103)^de^
WOG 1y	Control	1.00 (0.129)^a^	1.00 (0.183)^a^	1.00 (0.219)^a^	1.00 (0.117)^a^	1.00 (0.109)^ab^
	NaCl	0.84 (0.114)^ab^	0.74 (0.130)^abc^	0.84 (0.149)^ab^	0.88 (0.058)^ab^	1.18 (0.118)^a^
	Mannitol	0.40 (0.038)^d^	0.30 (0.020)^e^	0.30 (0.097)^d^	0.64 (0.085)^d^	0.89 (0.119)^bcd^
WOG 2y	Control	1.00 (0.081) ^a^	1.00 (0.179)^a^	1.00 (0.264)^a^	1.00 (0.201)^a^	1.00 (0.098)^ab^
	NaCl	0.82 (0.071)^ab^	0.74 (0.271)^abc^	0.71 (0.094)^ab^	0.98 (0.142)^a^	1.00 (0.201)^ab^
	Mannitol	0.48 (0.026)^cd^	0.34 (0.049)^de^	0.37 (0.054)^cd^	0.73 (0.103)^cd^	0.82 (0.089)^bcd^
WOG 3y	Control	1.00 (0.369)^a^	1.00 (0.241)^a^	1.00 (0.291)^a^	1.00 (0.286)^a^	1.00 (0.165)^ab^
	NaCl	1.02 (0.173)^a^	0.87 (0.301)^ab^	0.98 (0.086)^a^	1.01 (0.025)^a^	0.98 (0.135)^abc^
	Mannitol	0.53 (0.162)^cd^	0.55 (0.238)^cde^	0.81 (0.280)^ab^	0.95 (0.157)^ab^	1.20 (0.057)^a^

*Controls are normalized to the value 1. Different letters within the column present significant values at p < 0.05. Data are presented as averages (SD in parenthesis), n = 3. Raw data are presented in Supplementary Table 1*.

### Ion Content and Potassium Leakage in Olive Leaves Under NaCl- and Mannitol-Induced Stress

The uptake of ions in young leaves varied among olive cultivars and treatments (Table 3; Supplementary Table 2). In comparison with K^+^, the relative contents of Mg^2+^ and Ca^2+^ were more affected by both stressors in almost all genotypes. The mannitol treatment caused a greater reduction of contents of Mg^2+^ and Ca^2+^ in cv. Koroneiki and cv. Leccino than the NaCl treatment. However, both stressors caused the same degree of reduction in those nutrients in the WOG of different ages. The relative content of K^+^ in cv. Koroneiki decreased markedly under both treatments but especially under the mannitol treatment. It is interesting that the K^+^ content was not significantly affected compared with the control by NaCl or mannitol treatments in cv. Leccino or WOG of different ages (with the exception of 1-year-old WOG under the mannitol treatment). The highest values of KL were detected in the salt-treated WOG, especially 1-year-old WOG, while that parameter was not markedly influenced in cultivars.

**Table 3:** The relative ion content and K leakage of olive leaves.

**Genotype**	**Treatment**	**Mg**	**Ca**	**K**	**K leakage**
Koroneiki	Control	1.00 (0.029)^a^	1.00 (0.038)^a^	1.00 (0.029)^a^	1.00 (0.343)^c^
	NaCl	0.80 (0.010)^b^	0.77 (0.033)^cd^	0.71 (0.012)^c^	1.32 (0.422)^bc^
	Mannitol	0.62 (0.038)^e^	0.69 (0.009)^def^	0.59 (0.010)^d^	1.06 (0.439)^c^
Leccino	Control	1.00 (0.006)^a^	1.00 (0.049)^a^	1.00 (0.090)^a^	1.00 (0.167)^c^
	NaCl	0.75 (0.088)^bc^	0.88 (0.084)^b^	0.95 (0.039)^ab^	1.61 (0.205)^bc^
	Mannitol	0.62 (0.022)^e^	0.72 (0.090)^de^	0.93 (0.096)^ab^	1.34 (0.382)^bc^
WOG 1y	Control	1.00 (0.038)^a^	1.00 (0.063)^a^	1.00 (0.189)^a^	1.00 (0.305)^c^
	NaCl	0.66 (0.024)^de^	0.56 (0.016)^h^	1.03 (0.204)^a^	3.29 (0.369)^a^
	Mannitol	0.65 (0.031)^de^	0.61 (0.027)^fgh^	0.79 (0.056)^bc^	2.28 (0.809)^b^
WOG 2y	Control	1.00 (0.021)^a^	1.00 (0.057)^a^	1.00 (0.023)^a^	1.00 (0.362)^c^
	NaCl	0.69 (0.009)^cde^	0.67 (0.017)^efg^	0.98 (0.016)^a^	1.93 (0.891)^bc^
	Mannitol	0.64 (0.033)^de^	0.85 (0.066)^bc^	0.90 (0.006)^ab^	1.05 (0.263)^c^
WOG 3y	Control	1.00 (0.034)^a^	1.00 (0.138)^a^	1.00 (0.010)^a^	1.00 (0.109)^c^
	NaCl	0.64 (0.007)^de^	0.57 (0.004)^gh^	0.90 (0.001)^ab^	2.22 (0.808)^b^
	Mannitol	0.71 (0.079)^cd^	0.59 (0.057) ^gh^	0.93 (0.112)^ab^	1.99 (0.293)^bc^

*Controls are normalized to the value 1. Different letters within column present significant values at p < 0.05. Data are presented as averages (SD in parenthesis), n = 3. Raw data are presented in Supplementary Table 2*.

The relative contents of K^+^, Mg^2+^, and Ca^2+^ in olive roots are presented in Table 4; Supplementary Table 2a. With respect to root Mg^2+^, mannitol caused change only in the 1-year-old WOG and cv. Koroneiki, while NaCl decreased the content of this element in the 1- and 3-year-old WOG. The level of Ca^2+^ decreased in older WOG exposed to NaCl; otherwise, it was similar to control. A significant increase of Ca^2+^ was noted in mannitol-treated plants (except in 3-year-old WOG, where it was similar to the control). Root K^+^ showed a different trend than seen in shoots. A significant decline of root K^+^ was detected in treated cultivars and WOG, except in the 3-year-old WOG where K^+^ was similar to control.

**Table 4:** The relative ion content of olive roots.

**Genotype**	**Treatment**	**Mg**	**Ca**	**K**
Koroneiki	Control	1.00 (0.037)^bc^	1.00 (0.085)^c^	1.00 (0.042)^a^
	NaCl	0.99 (0.110)^bc^	0.85 (0.051)^c^	0.84 (0.019)^b^
	Mannitol	1.22 (0.061)^a^	1.85 (0.117)^a^	0.58 (0.010)^cd^
Leccino	Control	1.00 (0.033)^bc^	1.00 (0.020)^c^	1.00 (0.020)^a^
	NaCl	0.97 (0.217)^bc^	0.97 (0.208)^c^	0.47 (0.008)^de^
	Mannitol	0.92 (0.038)^bcd^	1.53 (0.101)^b^	0.49 (0.155)^de^
WOG 1y	Control	1.00 (0.054)^bc^	1.00 (0.061)^c^	1.00 (0.099)^a^
	NaCl	0.63 (0.051)^e^	0.81 (0.032)^cd^	0.343 (0.041)^f^
	Mannitol	0.77 (0.029)^de^	1.50 (0.125)^b^	0.378 (0.009)^bc^
WOG 2y	Control	1.00 (0.021)^bc^	1.00 (0.057)^c^	1.00 (0.023)^a^
	NaCl	0.85 (0.054)^cd^	0.63 (0.053)^de^	0.60 (0.019)^c^
	Mannitol	1.09 (0.165)^b^	1.44 (0.283)^b^	0.64 (0.026)^c^
WOG 3y	Control	1.00 (0.087)^bc^	1.00 (0.086)^c^	1.00 (0.033)^a^
	NaCl	0.67 (0.038)^e^	0.48 (0.038)^e^	0.89 (0.008)^ab^
	Mannitol	0.84 (0.057)^cd^	1.02 (0.077)^c^	0.89 (0.077)^ab^

*Controls are normalized on the value 1. Different letters within column present significant values at p < 0.05. Data are presented as averages (SD in parenthesis), n = 3. Raw data are presented in Supplementary Table 2a*.

**Figure 1:**
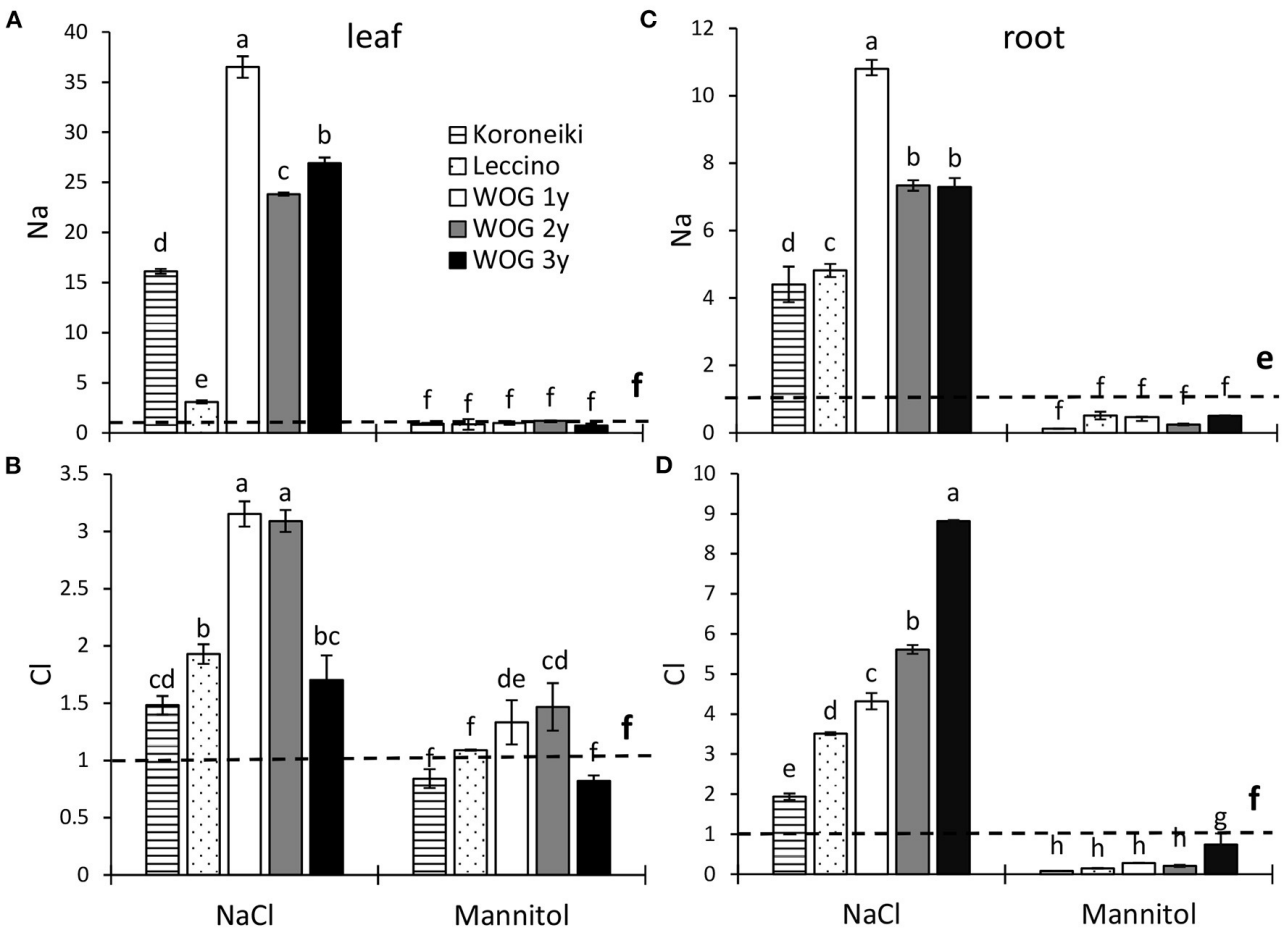
The relative contents of leaf Na^+^ **(A)**, leaf Cl^−^ **(B)**, root Na^+^ **(C)**, and root Cl^−^ **(D)** ions of olive after exposure to NaCl or mannitol. Controls are normalized to the value 1. Columns marked with different letters are statistically significantly different (*p* ≤ 0.05). The bars represent mean ±SD, *n* = 3. Raw data are presented in Supplementary Tables 2, 2a. SD, standard deviation.

Unlike mannitol, the NaCl treatment significantly affected the Na^+^ relative content in the leaves of all cultivars, particularly WOG (Figure 1A; Supplementary Table 2). The greatest accumulation of leaf Na^+^ was detected in 1-year-old WOG. The relative content of that ion was lower in older WOG (2- and 3-year-old) though still significantly increased compared with standard cultivars and control. The lowest Na^+^ relative content was detected in salt-sensitive cv. Leccino. The relative amount of Cl^−^ ion significantly increased with respect to the control in all cultivars treated with NaCl, particularly in the 1- and 2-year-old WOG (Figure 1B; Supplementary Table 2). With respect to the mannitol treatment, the micronutrient accumulated only in the 1- and 2-year-old WOG.

As in the leaf contents of salt ions, the level of Na^+^ and Cl^−^ in salt-treated olives was several times higher than in control plants (Figures 1C,D; Supplementary Table 2a). However, the highest contents of Na^+^ and Cl^−^ were recorded in WOG. It is interesting that accumulation of Na^+^ in olives, except in cv. Leccino, was far greater in the leaves than in the roots. Contrary to this, the Cl^−^ ions accumulated more in roots than in shoots. On the other hand, mannitol-treated plants showed lower levels of salt ions than in the control.

### Effects of NaCl and Mannitol on Biochemical Stress Parameters

**Figure 2:**
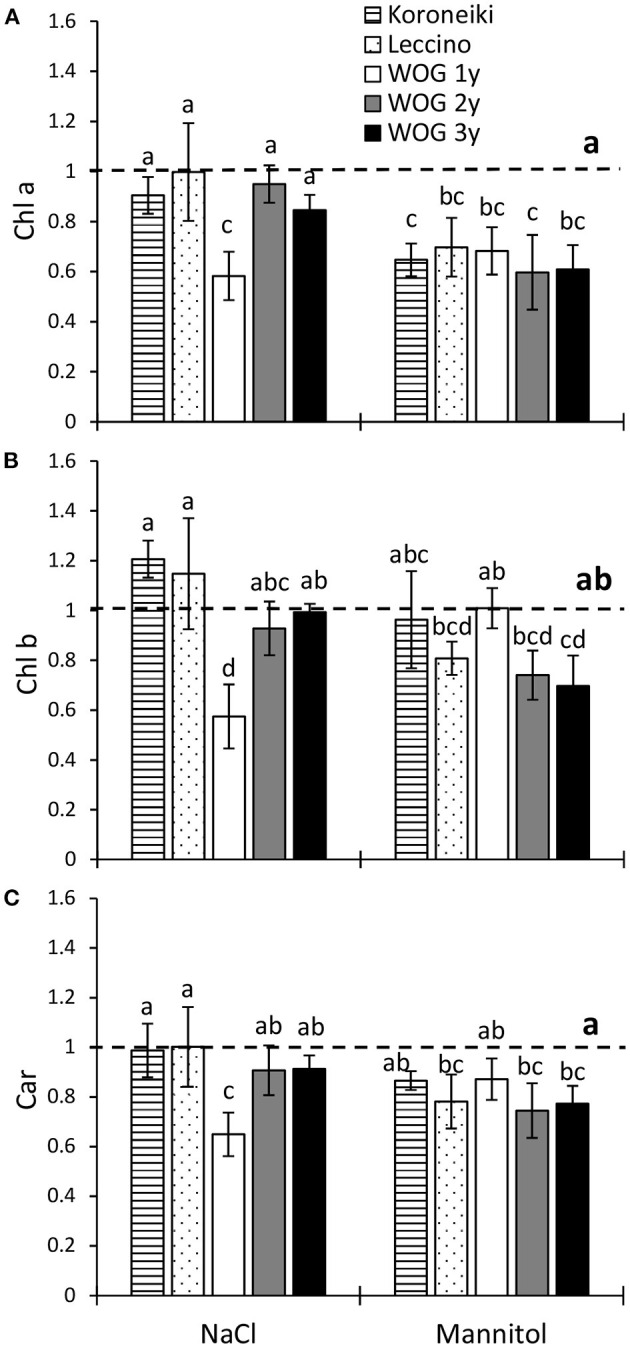
The relative contents of **(A)** chlorophyll a (Chl *a*), **(B)** chlorophyll *b* (Chl *b*), and **(C)** carotenoids (Car) in leaves of olive after exposure to NaCl or mannitol. Controls are normalized to the value 1. Columns marked with different letters are statistically significantly different (*p* ≤ 0.05). The bars represent mean ±SD, *n* = 3. Raw data are presented in Supplementary Table 3. SD, standard deviation.

The relative contents of measured photosynthetic pigments of all salt-treated plants were similar to the control (Figures 2A–C; Supplementary Table 3). The exception was the youngest WOG, where photosynthetic pigments significantly declined in response to NaCl. The mannitol treatment had a greater overall impact on photosynthetic pigments than NaCl, particularly on Chl *a*. Furthermore, a significant decrease of the relative Chl *a* content of all cultivars was detected under the mannitol treatment (Figure 2A), while Chl *b* was only reduced in the oldest WOG (Figure 2B). That osmoticum also caused a significant decline of total Car in cv. Leccino and in older WOG plants (2- and 3-year-old) compared with the control (Figure 2C).

**Figure 3:**
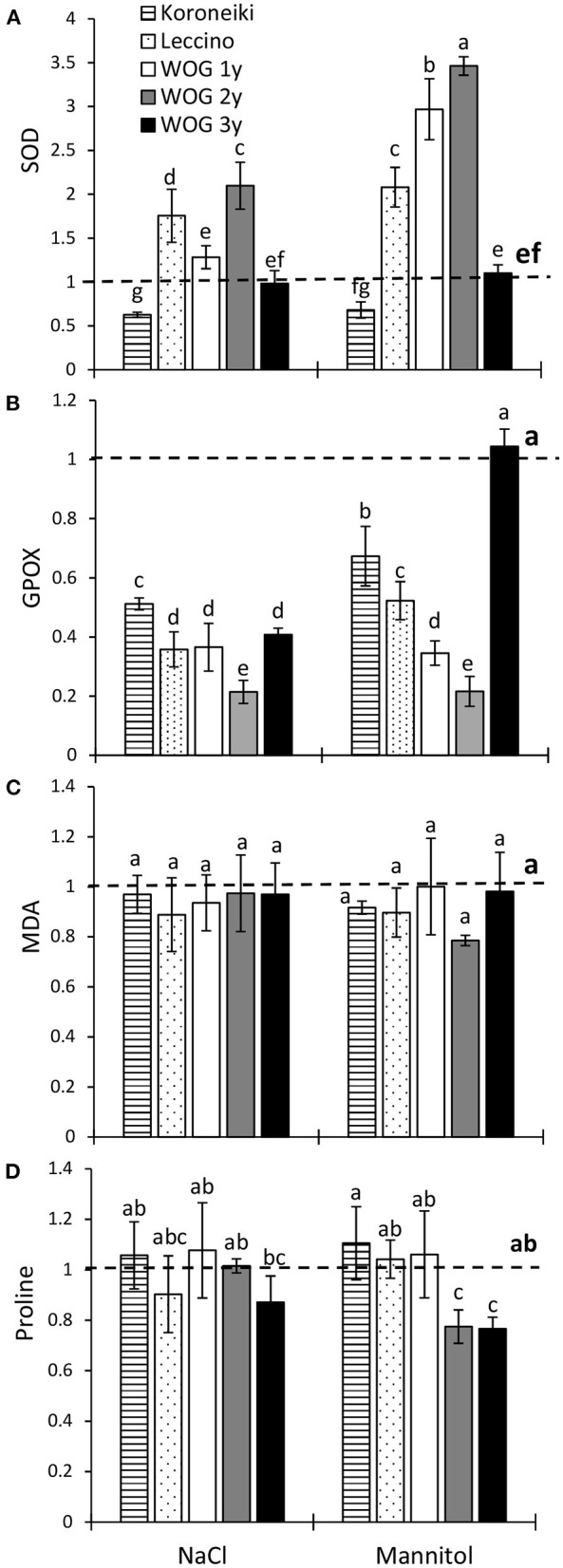
The relative activity of superoxide dismutase (SOD) **(A)** and guaiacol peroxidase (GPOX) **(B)**, relative contents of malondialdehyde (MDA) (C)and of proline **(D)** in olive leaves after exposure to NaCl or mannitol. Controls are normalized to the value 1. Columns marked with different letters are statistically significantly different (*p* ≤ 0.05). The bars represent mean ±SD, *n* = 3. Raw data are presented in Supplementary Table 3. SD, standard deviation.

In this study, the mannitol treatment induced the SOD activity in olive plants to a higher degree than the NaCl treatment (Figure 3A; Supplementary Table 3). The NaCl treatment significantly increased the relative activity of SOD of 2-year-old WOG and cv. Leccino in comparison with the control. The relative SOD activity of 1- and 2-year-old WOG under mannitol treatment was approximately three times higher than that of control plants, while the activity of the antioxidant enzyme doubled in cv. Leccino with respect to the control. It is interesting that the relative SOD activity of cv. Koroneiki was lower than that of control plants, regardless of the stressor. An inhibition of the GPOX activity was evident in all treated groups (Figure 3B), except in the oldest WOG where the activity of the enzyme reached the control level. The smallest decline of GPOX was noted in cv. Koroneiki. The MDA content did not vary between treatments or cultivars (Figure 3C); its level was similar to control in all treated plants.

Irrespective of the stressor, the levels of proline of almost all olive plants showed no statistical difference in comparison with the control (Figure 3D). Exceptions were observed in older WOG (i.e., 2- and 3-year-old) where the proline content was significantly lower than that in the control.

## Discussion

The results of the morphological, physiological, and biochemical analysis of WOG demonstrate the more severe effects of mannitol than NaCl. It was recognized decades ago that plants exposed to salinity first experience water stress (i.e., osmotic phase), due to which growth reduction becomes quickly apparent. After that phase, the salt accumulates in transpiring leaves, and the resulting ion toxicity causes a further decrease of growth (Munns, 2002). In this study, hyperosmotic stress induced by mannitol inhibited the growth of olive shoots on a much larger scale than NaCl-induced stress. Mannitol caused a decrease of shoot growth parameters in the WOG at the same level as in cv. Koroneiki and cv. Leccino. On the other hand, NaCl-induced salinity caused the greatest decline of shoot growth parameters in cv. Leccino, compared with cv. Koroneiki and WOG of different ages and also caused a decline of root DW of cv. Leccino. The result ties well with previous studies where cv. Leccino displayed the largest reduction in growth compared with the less vigorous cultivars (Perica et al., 2008; Rossi et al., 2014).

The difference between the effects of NaCl and mannitol on olive growth is likely related to the different osmotic adjustments. The use of salt ions for the osmotic adjustment is considered energetically more cost-effective than the biosynthesis of organic solutes, as long as the salt is sequestered in the vacuoles (Munns et al., 2020). When compartmentalization of salt ions in the vacuoles is no longer possible due to their excessive levels, ions then accumulate in the cytoplasm or cell walls, triggering a series of toxic effects (Munns, 2002). Here, the greatest accumulation of Na^+^ and a lesser degree of Cl^−^ were detected in the WOG leaves, although the cultivars also accumulated salt ions (Figure 1). After a short-term exposure to salinity, the 1-year-old WOG had the largest amounts of Na^+^ in leaves, followed by the same genotype of different ages and cv. Koroneiki, while cv. Leccino had the smallest amounts of that ion although the difference was still significant compared with the control. Compared with Na^+^, Cl^−^ accumulated at a lower level, though its content was highest in 1- and 2-year-old WOG. In previous reports, the highest amount of Na^+^ was usually determined in leaves of cv. Leccino compared with other cultivars, though in those studies, that cultivar was exposed to the long-term salt stress (Tattini et al., 1992; Perica et al., 2008; Tattini and Traversi, 2009; Rossi et al., 2014). Storey and Walker (1999) stated that certain salt-sensitive citrus genotypes take a longer time to accumulate salt ions to steady levels than salt-resistant genotypes. According to the analysis of salt ions in olive roots (Figure 1; Supplementary Tables 2, 2a), the exclusion of Na^+^ from the root to the sensitive leaf area was most efficient in cv. Leccino. Although WOG uses the same tolerance mechanism (Melgar et al., 2009; Fernández-Escobar, 2019), translocation of Na^+^ from root to shoot in these olives was far greater than in cv. Koroneiki and especially cv. Leccino. Since the growth of WOG shoots was not significantly affected, it seems that the compartmentalization of salt ions, Na^+^ in particular, in vacuoles is relatively efficient, and it serves to balance osmotic pressure in the leaf cells. In contrast to proline, Cl^−^ likely contributed to the osmotic adjustment of olive plants, though to a lesser extent than Na^+^. It has been reported that compared with Na^+^, olives are less sensitive to Cl^−^, which generally does not cause toxicity to olive trees (Tattini et al., 1992; Melgar et al., 2009). Regarding the mannitol treatment, the levels of leaf Na^+^ were similar to the control, while those of leaf Cl^−^ were higher in 1- and 2-year-old WOG compared with the control. The osmotic adjustment in the leaves of olive cultivars and WOG under hyperosmotic conditions could have been achieved by K^+^ and organic solutes other than proline, since no accumulation of that amino acid was observed in this study. Moreover, the levels of proline of all olive plants were either unchanged or even decreased in response to applied NaCl and mannitol (Figure 3C). Similar results were reported by Regni et al. (2019), whereas Ben Ahmed et al. (2009) detected an increase of proline in olives exposed to salinity. Since we did not measure the proline content in the roots of the tested olives, it cannot be claimed with certainty that proline is not engaged as an osmolyte in the roots of the tested olives. However, the levels of that amino acid in olive shoots do not support its role in the osmotic adjustment and adaptation to salt stress.

It has long been reported that salinity negatively influences the contents of nutrients in glycophytic crops, including olives (Loupassaki et al., 2002; Fernández-Escobar, 2019). In contrast to treated cv. Koroneiki, neither stressor affected the leaf K^+^ of cv. Leccino or WOG (Tables 3, 4; Supplementary Tables 2, 2a). This indicated that those olives were able to maintain leaf K^+^ content under stress conditions and likely use this ion in the cytoplasm to prevent water loss from the cell (Ragel et al., 2019). However, the content of root K^+^ of almost all olives decreased significantly. It is interesting that both NaCl and mannitol reduced the content of root K^+^ of the WOG to the same levels, regardless of age. This could imply that K uptake from the soil is restricted primarily by the osmotic component of the salt stress (Wang et al., 2013). Both stressors caused a decline of shoot Mg^2+^ and Ca^2+^ in all tested olives, though there was a difference between cultivars and WOG, where shoot levels decreased more under mannitol than the NaCl treatment in cultivars, while the degree of reduction of those nutrients was more conspicuous in WOG, and it was similar under both treatments. Among the tested olives, cv. Leccino had the highest amounts of Ca^2+^ in shoots under salinity treatment, while the root Ca^2+^ levels were either increased or remained unchanged under either treatment. Tattini and Traversi (2009) reported that salt-treated olive trees, which were additionally supplied with Ca^2+^, showed a superior ability to exclude Na^+^ from the shoot. We hypothesized that cv. Leccino, as a salt-sensitive olive cultivar, increased the influx of available Ca^2+^ from $\frac{1}{2}$ HNS once the salinity treatment was applied.

Electrolyte leakage (EL) is often used as an indicator of stress-related injury of plant tissues (Demidchik et al., 2014). In this study, EL of olives did not differ significantly between treatments (Table 1). The other parameter closely related to EL is KL, which refers to the efflux of K^+^ and certain counterions (Demidchik et al., 2014). Excessive KL was recorded in the 1-year-old WOG under saline stress, though this parameter was also markedly increased under hyperosmotic stress. Older WOG was less affected by either stressor compared with the 1-year-old WOG, which is likely associated with the plant age. Recent research implicates the involvement of ROS, in particular, hydroxyl radicals and H_2_O_2_, in the activation of K^+^ efflux channels (Demidchik et al., 2014). As the SOD activity of 1-year-old WOG was unchanged, we hypothesized that the greatest KL noticed in the leaves of 1-year-old WOG under saline stress may have resulted from the increased amounts of hydroxyl radicals and H_2_O_2_ generated by the activity of NADPH oxidase or from another source (Baxter et al., 2014).

Overall, mannitol had a stronger effect on photosynthetic pigments of all experimental olives than NaCl, especially on the content of Chl *a*. The salinity treatment significantly reduced the photosynthetic pigments only in the 1-year-old WOG (Figure 2). This suggests that the Chl *a* content of the youngest WOG is equally sensitive to the osmotic and ionic components of salt stress, whereas the contents of Chl *b* and Car are more affected by NaCl. However, photosynthetic pigments of older WOG to the stressors reached control levels, indicating their higher robustness to NaCl-induced stress, and also their higher sensitivity to hyperosmotic stress caused by mannitol. Contrary to our findings, the total chlorophyll content of the 7-month-old olive cv. Chétoui exposed to salinity and drought for 21 days was unaffected, while total carotenoids were also increased (Abdallah et al., 2018). However, in the study of Mousavi et al. (2019), a longer exposure (i.e., 43 days) of cv. Koroneiki to 200 mM NaCl caused a decrease of the chlorophyll content, although lower NaCl concentrations did not affect its values. This suggests that the response of olive plants to salinity is strongly dependent on the genotype, plant age, and stress duration (Fernández-Escobar, 2019; Henn and Damschen, 2021).

In general, SOD is the first line of defense against ROS, as that antioxidative enzyme catalyzes the dismutation of superoxide radicals mostly generated as a result of electron leakage from the photosynthetic and respiratory electron transport chains to oxygen (Sofo et al., 2008). The enzyme activity was increased in the 2-year-old WOG and cv. Leccino in response to the NaCl treatment, though a higher activity was seen in cv. Leccino and in 1- and 2-year-old WOG under mannitol-induced stress. However, neither stressor enhanced the SOD activity of the oldest WOG and cv. Koroneiki, suggesting their quicker adaptation to unfavorable conditions (Figure 3A). In the report of Goreta et al. (2007), the early response of cv. Leccino to salinity included the stimulation of the SOD activity, which declined later with a prolonged exposure to NaCl. In that study, an inverse pattern of change of the SOD activity with respect to stress duration was observed in the more salt-resistant cv. Oblica, implicating its more efficient mechanism of adaptation to salinity. However, it has to be noted that in the medium- and long-term salinity experiments, other signs of stress could emerge.

Hydrogen peroxide, a product of SOD, is further degraded by catalase and peroxidases (Das and Roychoudhury, 2014). Here, the inhibition of GPOX was seen in all plants under both treatments (except in 3-year-old WOG under the mannitol treatment), suggesting the enzyme was not active in scavenging of H_2_O_2_. The decreased activity of the enzyme was also recorded in olive cv. Arbequina exposed to the same salt concentration applied here, though for a longer time (Del Buono et al., 2021). Regardless of the stimulation of SOD, the decline of the GPOX activity, and increased KL in younger WOGs, the MDA content of those olives was similar to the control. Moreover, the absence of lipid peroxidation along with suppressed GPOX activity was noted in treated 3-year-old WOG and the cultivars. We hypothesized that some other enzymes, such as catalase and ascorbate peroxidase, could be involved in the detoxification of H_2_O_2_. In contrast to the results presented here, drought and salinity increased lipid peroxidation of cv. Chétoui after 21 days of exposure (Abdallah et al., 2018). In that study, plants were exposed to a higher salt concentration (200 mM), and drought stress was achieved by withholding irrigation, which could contribute to a higher level of oxidative stress.

Considering the influence of plant age on the level of resistance to salinity, the obtained results were not consistent, as certain growth and physiological traits significantly (e.g., photosynthetic pigments and root K^+^) or slightly improved (e.g., growth), while some others did not change or were even diminished (e.g., Mg^2+^ ion content) in older WOGs. Overall, it can be concluded that 2- and 3-year-WOG did not display a significantly better adaptive response to salinity. The reason for that could be that the difference in WOG age was too small to allow for a significant adaptive response to salt stress.

## Conclusion

New data on the response of wild olives to salinity are presented. Growth of WOG “Perišićeva mastrinka” was not significantly affected despite a considerable accumulation of salt ions in WOG leaves. The results show that the tolerance mechanisms of WOG “Perišićeva mastrinka” to salinity, under the experimental setting, include the utilization of salt ions (primarily Na^+^) for osmotic adjustment in WOG shoots, maintenance of required leaf K^+^ levels, and salt-stress responsive SOD. According to the results, the contribution of WOG age to better adaptive response to salinity was limited, at least in this age range (1–3 years). Overall, WOG has a promising potential for obtaining a new and better cultivar with favorable traits with respect to salt tolerance. Further experiments not only in the controlled conditions but in the field as well are required to investigate the long-term exposure of WOG to salinity and to compare its adaptive strategies to early responses.

## Data Availability Statement

The original contributions presented in the study are included in the article/Supplementary Material, further inquiries can be directed to the corresponding author.

## Author Contributions

JT and GD designed the research. JT carried the experiments of treatments and performed the morphological, physiological, and biochemical measurements and data analysis. MV performed the ion measurements and data analysis. SV performed the biochemical measurements and data analysis. SR conducted the statistical analysis and figure design. JT and SR drafted the manuscript. All authors contributed to manuscript revision and read and approved the submitted version.

## Conflict of Interest

The authors declare that the research was conducted in the absence of any commercial or financial relationships that could be construed as a potential conflict of interest.

## Publisher's Note

All claims expressed in this article are solely those of the authors and do not necessarily represent those of their affiliated organizations, or those of the publisher, the editors and the reviewers. Any product that may be evaluated in this article, or claim that may be made by its manufacturer, is not guaranteed or endorsed by the publisher.
