# Supplementary material for: Physiological and Biochemical Response of Wild Olive (Olea europaea Subsp. europaea var. sylvestris) to Salinity
Source: Front Plant Sci. 2021 Aug 30;12:712005. doi: 10.3389/fpls.2021.712005 (PMC8437259; doi:10.3389/fpls.2021.712005)
Supplement: Supplementary file 1 [file Data_Sheet_1.docx]

Figure S1. EC value of treatments during the experiment (control - 1/2 Hoagland solution; Mannitol - 1/2 Hoagland solution + 300 mM Mannitol ; NaCl - 1/2 Hoagland solution + 150 mM NaCl).
