# Supplementary figures and images for: Physiological and Biochemical Response of Wild Olive (Olea europaea Subsp. europaea var. sylvestris) to Salinity
Source: Front Plant Sci. 2021 Aug 30;12:712005. doi: 10.3389/fpls.2021.712005 (PMC8437259; doi:10.3389/fpls.2021.712005)

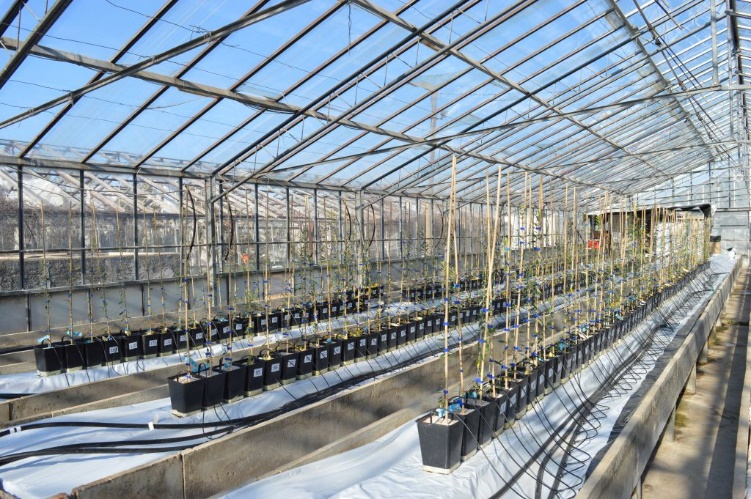


Figure S2: Olive plants in greenhouse (photo: J Tadić).

Supplement: Supplementary file 2 [file Data_Sheet_2.docx]
